# Exercise capacity and physical activity in COPD patients treated with a LAMA/LABA combination: a systematic review and meta-analysis

**DOI:** 10.1186/s12931-022-02268-3

**Published:** 2022-12-15

**Authors:** Marc Miravitlles, Juan Luís García-Rivero, Xavier Ribera, Jordi Galera, Alejandra García, Rosa Palomino, Xavier Pomares

**Affiliations:** 1 Pneumology Department, Hospital Universitari Vall d’HebronVall d’Hebron Institut de Recerca, Vall d’Hebron Barcelona Hospital Campus, Pg. Vall d’Hebron 119-129, 08035 Barcelona, Spain; 2 Pneumology Department, President of ACINAR, Hospital Universitario Marqués de Valdecilla, Santander, Spain; 3 Boehringer Ingelheim España S.A., Barcelona, Spain; 4 TFS Health Science, Barcelona, Spain; 5 Market Access AreaPharmalex Spain, Barcelona, Spain; 6 Pneumology Department, Hospital de Sabadell, Hospital Universitari Parc TaulíInstitut Investigació i Innovació Parc Taulí I3PT, Universitat Autònoma de Barcelona, Sabadell, Spain

**Keywords:** Bronchodilators, COPD, Exercise capacity, LABA, LAMA, Physical activity

## Abstract

**Background:**

Persistent airflow limitation and dyspnoea may reduce chronic obstructive pulmonary disease (COPD) patients exercise capacity and physical activity, undermining their physical status and quality of life. Long-acting muscarinic antagonists and long-acting beta-2 agonists (LAMA/LABA) combinations are amongst moderate-to-severe COPD recommended treatments. This article analyses LAMA/LABA combinations effect on COPD patients exercise capacity and physical activity outcomes.

**Methods:**

A systematic review and meta-analysis of double-blind randomized controlled trials comparing LAMA/LABA combinations against monotherapy or placebo was conducted.

**Results:**

Seventeen articles were identified (N = 4041 patients). In endurance shuttle walk test and constant work rate cycle ergometry, LAMA/LABA combinations obtained better results than placebo, but not monotherapy, whereas in 6-min walking test, results favoured LAMA/LABA over monotherapy (four studies), but not over placebo (one study). Moreover, LAMA/LABA combinations obtained better results than placebo in number of steps per day, reduction in percentage of inactive patients and daily activity-related energy expenditure, and better than monotherapy when measuring time spent on ≥ 1.0–1.5, ≥ 2.0 and ≥ 3.0 metabolic equivalents of task activities.

**Conclusions:**

LAMA/LABA combinations in COPD patients provided better results than monotherapy or placebo in most exercise capacity and physical activity outcomes.

**Supplementary Information:**

The online version contains supplementary material available at 10.1186/s12931-022-02268-3.

## Background

Patients with chronic obstructive pulmonary disease (COPD) present persistent airflow limitation and dyspnoea that result in reduced exercise capacity and/or physical activity, which can undermine their physical status and quality of life [1, 2]. Physical inactivity is the most important predictor of all-cause mortality in COPD and inactivity by itself induces a higher physical deterioration creating a vicious circle that results in isolation and increased mortality [3, 4]. A study carried out in Latin America showed that low levels of physical activity are especially important in women and older patients, and it is related with worse functional and clinical factors [4]. Therefore, the Global Initiative for Chronic Obstructive Lung Disease (GOLD) recommends regular physical activity for COPD patients [1]. The documented reduction in daily activity in COPD patients results from the respiratory and non-respiratory clinical conditions of each patient. Particularly, the limitation on exercise capacity is mainly due to dynamic pulmonary hyperinflation, although other factors also contribute, such as comorbidities or an imbalance between respiratory and locomotive muscles due to limited energy supply [5]. Moreover, it has been shown that the exercise capacity and the limitation in daily activities are closely related to life expectancy and, therefore, pulmonary and systemic manifestations would be improved by improving exercise capacity and physical activity [6].

The recommended treatment for patients with moderate-to-severe COPD and for symptomatic patients or those with exercise limitation, is inhaled long-acting beta-2 agonists (LABA) and/or long-acting muscarinic antagonists (LAMA) [1, 6, 7]. Bronchodilators increase lung emptying by reducing airway resistance, enabling COPD patients to achieve better alveolar ventilation with a lower operating pulmonary volume, both at rest and during exercise. As a result, patients using bronchodilators are able to exercise for longer before reaching the critical limit of their inspiratory reserve [8].

Due to the relevance of exercise capacity and physical activity on the quality of life of COPD patients, we have conducted a systematic literature review (SLR) and meta-analysis of randomized clinical trials aimed to evaluate the effect of the combination of LAMA/LABA bronchodilators compared with placebo or LAMA or LABA monotherapy on the exercise capacity and physical activity outcomes of COPD patients.

## Methods

This SLR was carried out according to the Preferred Reporting Items for Systematic Reviews and Meta-analyses Statement (PRISMA) and the QUORUM Statement [9]. The protocol was registered with PROSPERO (CRD42020191639).

### Inclusion and exclusion criteria

We included randomized clinical trials in patients aged ≥ 40 years diagnosed with COPD, with a post-bronchodilator forced expiratory volume at 1 s (FEV_1_)/forced vital capacity (FVC) < 0.7 and treated with a combination of LAMA/LABA inhaled bronchodilators compared with placebo or monotherapy with LAMA or LABA. To be included, the trials had to evaluate at least one variable related to exercise capacity or physical activity.

### Search strategy

A search strategy was designed for MEDLINE (through PubMed), CENTRAL and EMBASE using appropriate controlled terms related to COPD, LAMA, LABA, exercise capacity, physical activity and lung function in articles published between the 1st January 2012 and the 31st December 2021 as the first LAMA/LABA combination inhaler was approved in 2013 and prior 2012 there was no evidence about double bronchodilators (Additional file 2: Table A1). There were no limitations regarding language. Additionally, references to selected articles were also reviewed to identify other articles that met the inclusion and exclusion criteria.

### Study selection and data extraction

The titles and abstracts resulting from the search were evaluated by two reviewers. The studies that didn’t meet inclusion and exclusion criteria were ruled out, collecting the reasons for exclusion. The articles selected were read independently in full by the same two reviewers, who recorded the reasons for non-selection. In the event of discrepancies between the reviewers, the criterion of a third reviewer was used.

Data from the selected articles were tabulated by one reviewer and validated by a second reviewer in a detailed extraction form. From each article we extracted the study characteristics (type of study, design, countries), patient characteristics (mean age, sex, disease severity), and interventions and comparators (LAMA/LABA, LAMA, LABA or placebo inhalers used, dose, treatment duration), and the results of variables related to the exercise capacity and physical activity.

### Assessed outcomes

The identified outcome variables are defined as:

- 6-min walking test (6MWT), measuring the distance walked in 6 min in meters.
- Endurance Shuttle Walk Test (ESWT) measured in seconds (one study measured it in mean percentage change from baseline).
- Constant Work Rate Cycle Ergometry (CWRCE) measured in seconds.
- Steps per day (steps/day), examined by accelerometer and evaluated as average number of steps per day.
- Energy expenditure of ≥ 1.0–1.5, ≥ 2.0, ≥ 3.0 Metabolic Equivalent of Task (METs), consisting on the average daily duration (in minutes) of ≥ 1.0–1.5, ≥ 2.0 and ≥ 3.0 METs. Periods of sedentary time were categorized as an energy expenditure of 1.0–1.5 METs, whereas periods of physical activity performed at more than light (i.e., moderate, or vigorous) and more than moderate (i.e., vigorous) intensities were categorized as ≥ 2.0 METs and ≥ 3.0 METs, respectively.
- Energy expenditure related to activity, measured in kilocalories per day.
- Walking time per day, measured in minutes per day.
- Walking intensity, average daily walking intensity measured in meters per square second.
- Percentage of inactive patients, where inactive patient was defined as patient who walked less than 6,000 steps per day.
- Daily PROactive Physical Activity COPD questionnaire (D-PPAC) punctuation (questionnaire punctuation) is a daily recall, electronic, patient-reported outcome (PRO) tool, it was filled out by patients every evening for a period of time (usually a week). This seven-item PRO measure consists of two physical activity experience domains: amount and difficulty [10].

### Assessment of risk of bias

The risk of bias assessment was carried out according to the Cochrane Manual for Systematic Reviews and Meta-Analysis of Interventions criteria [11] and evaluated the generation of the randomization sequence, concealment of the assignment, blinding of patients and researchers, blinding of the results of the variables to be evaluated, data on incomplete results, bias of scientific information, and other biases. The risk of bias was assessed by one reviewer and validated by a second on a detailed form. Review Manager 5.4 was used for the risk of bias assessment.

### Data analysis

The analysis was based on the change from baseline in the above-mentioned outcome variables and assessed using dichotomous and continuous outcomes. Dichotomous data were analysed by calculating the estimate for the odds ratio (OR) and their corresponding 95% confidence intervals (CI). Continuous data were analysed by calculating weighted mean differences (WMD) and standardized mean differences (SMD), both with the corresponding 95% CI.

When useful, forest plots were created, in order to graphically assess the variability of sample estimates and the weight of sample sizes in the calculation of estimates (weighted averages). In addition, to facilitate interpretation of the results from studies that were not included in the forest plots, the mean and standard deviation were shown. A significance level of α = 0.05 was considered.

For data synthesis among studies, statistical heterogeneity was evaluated using I^2^, with I^2^ > 50% considered to be significant heterogeneity. In those comparisons with no statistical evidence of heterogeneity, a fixed effects model was used; otherwise, a random effects model was employed.

A sensitivity analysis stratified by study design (parallel and cross-over) was performed for results that showed heterogeneity (I^2^ > 0%).

The analysis considered the results of two treatment arms compared in each study. For studies with more than 2 treatment arms, comparisons were made separately, dividing the sample size of the study by the number of comparisons to avoid overestimation of results. The analysis was made using Review Manager 5.4.

## Results

**Fig. 1:**
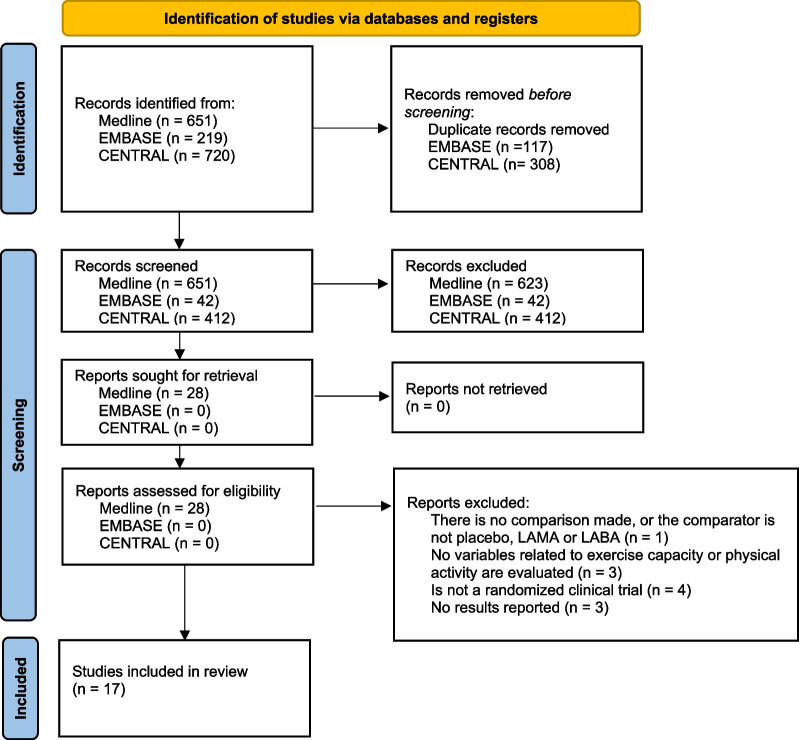
PRISMA flowchart. *COPD* chronic obstructive pulmonary disease, *LABA* long-acting beta-2 agonists, *LAMA* long-acting muscarinic antagonists, *PRISMA* Preferred Reporting Items for Systematic Reviews and Meta-analyses Statement

The search strategies yielded 1590 articles, of which 17, including 4,041 patients, met the inclusion criteria, 2964 of the patients were treated with the LAMA/LABA combination, 1901 treated with placebo, 1070 treated with LAMA and 755 treated with LABA (Fig. 1) [12–28]. The reference search yielded no further articles.

### Description of the studies

All included studies were randomized, controlled, double-blind trials. Five studies had a parallel design, including between 80 and 404 participants and the remaining twelve were crossover trials, including 17–657 participants (mean 238, median 184). The duration of treatment was 52 weeks (1 study), 12 weeks (7 studies), 8 weeks (1 study), 6 weeks (4 studies), 4 weeks (1 study), 3 weeks (1 study) and 2 weeks (1 study) (Table 1).

**Table 1:** Characteristics of studies included

Abbreviated reference and countries	Study type, number of arms and randomized patients	Duration	Inclusion criteria	Intervention	Comparator	Outcomes selected for analysis	Main results
Troosters et al. 2018 [12] Australia, Austria, Belgium, Canada, Denmark, Germany, New Zealand, Poland, Portugal, United Kingdom, United States	Parallel, 4 arms, 304 patients	12 weeks	40–75 years, smoking history > 10 pack-years, FEV1 post-bronchodilator 30% to 80% predicted, and FEV1/FVC < 70%	Tiotropium 5 μg/Olodaterol 5 μg, or Tiotropium 5 μg/Olodaterol 5 μg with training program	Tiotropium 5 μg or placebo	ESWT, 6MWT, steps/day, walking intensity, walking time/day	Arithmetic mean (SE) change from baseline, ESWT: - Tiotropium 5 μg/Olodaterol 5 μg: 91.0 (28.0) - Placebo: 31.0 (30.0) Adjusted mean (SE) change from baseline, 6MWT: - Tiotropium 5 μg/Olodaterol 5 μg: 25.76 (7.17) - Tiotropium 5 μg: 6.87 (7.75) - Placebo: 13.89 (8.09) Adjusted mean (SE) change from baseline, steps/day: - Tiotropium 5 μg/Olodaterol 5 μg: 1394.24 (310.22) - Tiotropium 5 μg: 153.19 (317.98) - Placebo: 1098.07 (325.08) Walking intensity and walking time/day results showed in results section
O’Donnell et al. 2017 [13] United States, Argentina, Australia, Austria, Belgium, Canada, Chile, Germany, Italy, Netherlands, New Zealand, Russia, Sweden	Crossover 5 arms, 586 patients	6 weeks	Smoking history > 10 pack-years, post-bronchodilator FEV1/FVC < 0.7; post-bronchodilator FEV1 ≥ 30% and < 80% of predicted	Tiotropium 2.5 g/Olodaterol 5 μg, or Tiotropium 5 μg/Olodaterol 5 μg	Tiotropium 5 μg, olodaterol 5 µg and placebo	CWRCE	Adjusted arithmetic mean (SE) of EET during CWRCE: - At Beginning: - All treatments: 511.6 (SD: 269.4) - At 6 weeks: - Placebo: 470.6 (12.6) - Olodaterol 5 μg: 521.1 (12.6) - Tiotropium 5 μg: 536.2 (12.6) - Tiotropium 2.5 g/Olodaterol 5 μg: 552.1 (12.5) - Tiotropium 5 μg/Olodaterol 5 μg: 554.5 (12.5)
Ichinose et al. 2018 [14] Japan	Crossover 2 arms, 184 patients	6 weeks	Japanese patients ≥ 40 years, with history of smoking > 10 pack-years, with COPD and stable airway obstruction, post-bronchodilator FEV1 < 80% of predicted; post-bronchodilator FEV1/FVC < 0.7 at visit 1; mMRC ≥ 1; 6MWD < 400 m; and a score ≥ 4 on Borg's modified scale following the 6MWD test on visit 2	Tiotropium 5 μg/Olodaterol 5 μg	Tiotropium 5 μg	6MWT, steps/day, duration of activity	Adjusted mean (SD), 6MWT: - At Beginning: - All treatments: 293.8 (93.3) - At 6 weeks: - Tiotropium 5 μg/Olodaterol 5 μg: 311.5 (n.a) - Tiotropium 5 μg: 307.4 (n.a) Adjusted mean (95%CI) treatment difference at 6 weeks, 6MWT: 4.2 (-6.2–14.5) Adjusted mean, steps/day: - At Beginning: - All treatments: 3723.0 - At 6 weeks: - Tiotropium 5 μg/Olodaterol 5 μg: 3871.1 - Tiotropium 5 μg: 3793.6 Adjusted mean (95%CI) treatment difference at 6 weeks, steps/day: 77.5 (-92.7–247.7) Adjusted mean (SD), ≥ 2 METs: - At Beginning: - All treatments: 181.4 (82.0) - At 6 weeks: - Tiotropium 5 μg/Olodaterol 5 μg: 191.5 (n.a) - Tiotropium 5 μg: 186.5 (n.a) Adjusted mean (95%CI) treatment difference at 6 weeks, ≥ 2 METs: 5.0 (0.39–9.69)
Watz et al. 2017 [15] Canada, Germany, Hungary, Spain	Parallel 2 arms, 267 patients	8 weeks	≥ 40 years, history of smoking, (FRC) ≥ 120% of predicted, post-bronchodilator FEV1 ≥ 40% and < 80% of predicted, FEV1/FVC < 70%, and score ≥ 2 on mMRC dyspnoea scale	Aclidinium 800 μg/Formoterol 24 μg	Placebo	CWRCE, % inactive patients, steps/day, duration of activity, energy expenditure, D-PPAC	Least square mean (95%CI) change from baseline, CWRCE: - Aclidinium 800 μg/Formoterol 24 μg: 45.5 (13.7–77.3) - Placebo: -13.40 (n.a) Treatment difference of % inactive patients: OR:0.27; 95%CI: 0.14–0.51 Least squares mean (SE) change from baseline, steps/day: - Aclidinium 800 μg/Formoterol 24 μg: 621.0 (167.0) - Placebo: -110.0 (167.0) Least squares mean (95%CI) change from baseline, ≥ 3 METs: - Aclidinium 800 μg/Formoterol 24 μg: 8.5 (4.3–12.8) - Placebo: -1.2 (-5.40–3.10) Adjusted mean (95%CI) treatment difference at 4 weeks, ≥ 3 METs: 9.7 (3.8–15.5) Least squares mean (95%CI) change from baseline, energy expenditure: - Aclidinium 800 μg/Formoterol 24 μg: 36.5 (16.8–56.1) - Placebo: − 4.4 (− 24.1 to 15.2) Adjusted mean (CI-95%) treatment difference at 4 weeks, energy expenditure: 40.9 (13.9–67.9) D-PPAC results showed in results section
Minakata et al. 2019 [16] Japan	Crossover 2 arms, 182 patients	6 weeks	≥ 40 years, diagnosed with COPD and GOLD grade II–IV	Tiotropium 5 μg/Olodaterol 5 μg	Tiotropium 5 μg	MET(s)	Adjusted mean (SD), ≥ 1.0–1.5 METs: - At Beginning: - All treatments: 408.4 (90.0) - At 6 weeks: - Tiotropium 5 μg/Olodaterol 5 μg: 407.9 (n.a) - Tiotropium 5 μg: 416.6 (n.a) Adjusted mean (95%CI) treatment difference at 6 weeks, ≥ 1.0–1.5 METs: -8.64 (-16.88–-0.40) Adjusted mean (SD), ≥ 2 METs: - At Beginning: - All treatments: 177.30 (64.4) - At 6 weeks: - Tiotropium 5 μg/Olodaterol 5 μg: 179.1 (n.a) - Tiotropium 5 μg: 172.5 (n.a) Adjusted mean (95%CI) treatment difference at 6 weeks, ≥ 2 METs: 6.51 (1.17–11.85) Adjusted mean (SD), ≥ 3 METs: - At Beginning: - All treatments: 42.2 (24.7) - At 6 weeks: - Tiotropium 5 μg/Olodaterol 5 μg: 46.1 (n.a) - Tiotropium 5 μg: 43.5 (n.a) Adjusted mean (95%CI) treatment difference at 6 weeks, ≥ 3 METs: 2.6 (0.7–4.49)
Singh et al. 2018 [17] United States, Bulgaria, Estonia, Germany, Russia, United Kingdom, Canada, Czech Republic, Denmark, South Africa, Ukraine	Crossover 4 arms, 657 patients	12 weeks	≥ 40 years, with history of smoking > 10 pack-years, FRC at rest > 120% of predicted, FEV1/FVC post-bronchodilator < 70% and FEV1 ≥ 35% and ≤ 70% of predicted	Umeclidinium 62.5 μg/Vilanterol 25 μg	Umeclidinium 62.5 μg, Vilanterol 25 μg, and	ESWT	Least squares mean (SE) change from baseline, ESWT: - Umeclidinium 62.5 μg/ Vilanterol 25 μg: 27.3 (4.4) - Umeclidinium 62.5 μg: 20.4 (7.7) - Vilanterol 25 μg: 12.6 (6.3)
Riley et al. 2018 [18] United States	Crossover 2 arms, 198 patients	12 weeks	≥ 40 years, history of smoking ≥ 10 packages/year; FEV1/FVC < 0.70 and FEV1 post-bronchodilation 30–70% of predicted; FRC at rest ≥ 120% of predicted; score ≥ 2 on mMRC dyspnoea scale	Umeclidinium 62.5 μg/Vilanterol 25 μg	Placebo	ESWT	Least squares mean (SE) change from baseline, ESWT - Umeclidinium 62.5 μg/ Vilanterol 25 μg: -2.1 (9.29) - Placebo: − 5.4 (9.68)
O’Donnell et al. 2018 [19] Canada	Crossover 2 arms, 17 patients	4 weeks	> 40 years, with smoking history > 20 pack-years, post-bronchodilator FEV1 ≥ 50 and < 80% over predicted, FEV1/FVC < 0.7, and activity-related dyspnoea (BDI ≤ 9 or mMRC dyspnoea scale ≥ 2)	Umeclidinium 125 μg/Vilanterol 25 μg	Umeclidinium 125 μg	CWRCE	Mean (SD), CWRCR (min): - At Beginning: - Umeclidinium 125 μg/Vilanterol 25 μg: 7.19 (4.13) - Umeclidinium 125 μg: 6.83 (4.67) - At 4 weeks: - Umeclidinium 125 μg/Vilanterol 25 μg: 7.49 (4.99) - Umeclidinium 125 μg: 7.82 (6.15)
Maltais et al. 2018 [20] United States, Argentina, Canada, Finland, France, Germany, Hungary, Italy, Spain, United Kingdom	Parallel 3 arms, 404 patients	12 weeks	40–75 years, with history of smoking > 10 pack-years; post-bronchodilator FEV1/FVC < 70% and post-bronchodilator FEV1 < 80% and ≥ 30% above predicted	Tiotropium 2.5 μg/Olodaterol 5 μg, or Tiotropium 5 μg/Olodaterol 5 μg	Placebo	CWRCE, ESWT	Adjusted arithmetic mean (SD) of EET during CWRCE: - At Beginning: - Tiotropium 2.5 μg/Olodaterol 5 μg: 490.7 (272.4) - Tiotropium 5 μg/Olodaterol 5 μg: 527.5 (279.2) - Placebo: 502.7 (258.6) - At 12 weeks: - Tiotropium 2.5 μg/Olodaterol 5: 616.35 (SE: 23.41) - Tiotropium 5 μg/Olodaterol 5 μg: 628.32 (SE: 22.94) - Placebo: 549.42 (SE:24.36) Adjusted arithmetic mean (SD) of EET during CWRCE: - At Beginning: - Tiotropium 2.5 μg/Olodaterol 5: 366.7 (206.0) - Tiotropium 5 μg/Olodaterol 5 μg: 373.7 (217.1) - Placebo: 346.3 (186.5) - At 12 weeks: - Tiotropium 2.5 μg/Olodaterol 5: 473.43 (SE: 31.47) - Tiotropium 5 μg/Olodaterol 5 μg: 465.48 (SE: 30.40) - Placebo: 379.95 (SE:33.06)
Maltais et al. 2014 [28] United States, Bulgaria, Estonia, Germany, Russia, United Kingdom, Canada, Czech Republic, Denmark, South Africa, Ukraine	Crossover 6 arms, 655 patients	12 weeks	≥ 40 years, with history of smoking ≥ 10 pack-years, post-bronchodilator FEV1/FVC < 70% and FEV1 ≥ 35% and ≤ 70% of predicted; score ≥ 2 on mMRC dyspnoea scale on visit 1 and FRC at rest ≥ 120% of predicted	Umeclidinium 62.5 μg/ Vilanterol 25 μg or Umeclidinium 125 μg/ Vilanterol 25 μg	placebo	ESWT	Adjusted arithmetic mean (SE) of EET during ESWT, change from baseline: - Umeclidinium 62.5 μg/ Vilanterol 25 μg: 62.9 (10.8) - Umeclidinium 125 μg/ Vilanterol 25 μg: 66.7 (10.99) - Placebo: 19.2 (10.39)
Watz et al. 2016 [21] Germany	Crossover 2 arms, 194 patients	3 weeks	≥ 40 years, with history of smoking ≥ 10 pack-years, post-bronchodilator FEV1 between 40 and 80% of predicted and FEV1/FVC < 0.70 at visit 2	Indacaterol 110 μg/ Glycopyrronium 50 μg	Placebo	Energy expenditure, steps/day, duration of activity	Least squares mean change from baseline, energy expenditure: - Indacaterol 110 μg/Glycopyrronium 50 μg: 5.10 - Placebo: − 31.60 Least squares mean (95%CI) treatment difference, change from baseline, energy expenditure: 36.7 (1.7–71.7) Mean (SD) change from baseline, steps/day: - Indacaterol 110 μg/Glycopyrronium 50 μg: 31.0 (1662.4) - Placebo: − 321.0 (1647.6) Mean (SD) treatment difference, change from baseline, steps/day: 358.0 (2458.0) Least squares mean (95%CI) change from baseline, ≥ 3 METs: - Indacaterol 110 μg/Glycopyrronium 50 μg: − 6.9 (− 13.4 to − 0.40) - Placebo: − 11.3 (− 17.9 to − 4.60) Least squares mean (95%CI) treatment difference, change from baseline, ≥ 3 METs: 4.4 (-3.30–12.1)
Maltais et al. 2020 [22] United States, Argentina, Canada, Finland, France, Germany, Hungary, Italy, Spain, United Kingdom	Parallel 3 arms, 151 patients	12 weeks	40–75 years, post-bronchodilator FEV1 between ≥ 30% and < 80% than predicted, and post-bronchodilator FEV1/FVC < 70%	Tiotropium 2.5 μg/Olodaterol 5 μg, or Tiotropium 5 μg/Olodaterol 5 μg	Placebo	CWRCE, ESWT	Arithmetic mean CWRCE (SE) at week 6: - Placebo: 425.2 (25.3) - Tiotropium 5 μg/Olodaterol 5 μg: 507.0 (27.0) Arithmetic mean ESWT (SE) at 6 weeks6: - Placebo: 375.6 (34.0) - Tiotropium 5 μg/Olodaterol 5 μg: 457.2 (30.3)
Canto et al. 2012 [23] Brazil	Crossover 2 arms, 41 patients	2 weeks	Patients with stable COPD who met GOLD criteria, with a history of smoking > 20 pack-years	Formoterol 24 μg /Tiotropium 18 μg	Placebo/Formoterol 12 μg	Tolerance limit in constant work rate test	Percentage of mean (SD) change from baseline: - Formoterol 24 μg /Tiotropium 18 μg: 84.5 (8.2) - Placebo/Formoterol 12 μg: 40.7 (7.6)
Jayaram et al. 2013 [24] Australia, New Zealand	Crossover 2 arms, 38 patients	6 weeks	Age: 18–80 years, smoking history ≥ 10 pack-years, COPD defined by ATS/ERS criteria	Formoterol 24 μg /Tiotropium 18 μg	Placebo /Tiotropium 5 μg	6MWT	Mean (95%CI) change from baseline, 6MWT: - Formoterol 24 μg /Tiotropium 18 μg: 25.5 (4.4–46.5) - Placebo /Tiotropium 5 μg: − 7.6 (− 23.1 to 7.8) Mean (CI-95%) treatment difference at 6 weeks, ≥ 2 METs: 36.3 (2.4–70.1)
Takahashi et al. 2020 [25] Japan	Parallel, 2 arms, 80 patients	12 weeks	40 to 85 years, untreated, with smoking history ≥ 10 packages/year, post-bronchodilator FEV1 < 80% predicted, and FEV1/FVC < 70%	Tiotropium 5 μg/Olodaterol 5 μg	Tiotropium 5 μg	6MWT, steps/day, MET(s)	Mean (SD), 6MWT: - At Beginning: - Tiotropium 5 μg/Olodaterol 5 μg: 470.3 (77.6) - Tiotropium 5 μg: 438.8 (88.1) - At 12 weeks: - Tiotropium 5 μg/Olodaterol 5 μg: 475.7 (68.7) - Tiotropium 5 μg: 445.7 (80.6) Mean (SE) change from baseline, steps/day: - Tiotropium 5 μg/Olodaterol 5 μg: 168.1 (392.5) - Tiotropium 5 μg: 37.6 (192.4) Mean (CI-95%) treatment difference at 6 weeks, steps/day: 130.5 (− 750.0 to 1011.1) Mean (SD), ≥ 1.0–1.5 METs: - At Beginning: - Tiotropium 5 μg/Olodaterol 5 μg: 299.6 (92.4) - Tiotropium 5 μg: 287.0 (97.1) - Change from baseline: - Tiotropium 5 μg/Olodaterol 5 μg: -38.7 (n.a) - Tiotropium 5 μg: − 4.6 (n.a) Mean (95%CI) treatment difference at 12 weeks, ≥ 1.0–1.5 METs: -34.1 (-70.4–2.2) Mean (SD), ≥ 2 METs: - At Beginning: - Tiotropium 5 μg/Olodaterol 5 μg: 138.5 (63.3) - Tiotropium 5 μg: 141.2 (68.5) - Change from baseline: - Tiotropium 5 μg/Olodaterol 5 μg: 10.8 (n.a) - Tiotropium 5 μg: 8.3 (n.a) Mean (95%CI) treatment difference at 12 weeks, ≥ 2 METs: 2.5 (− 19.0 to 24.0) Mean (SD), ≥ 3 METs: - At Beginning: - Tiotropium 5 μg/Olodaterol 5 μg: 41.0 (29.0) - Tiotropium 5 μg: 36.1 (24.2) - Change from baseline: - Tiotropium 5 μg/Olodaterol 5 μg: 5.2 (n.a) - Tiotropium 5 μg: 2.5 (n.a) Mean (95%CI) treatment difference at 12 weeks, ≥ 3 METs: 2.7 (-7.4–12.8)
Stringer et al. 2021 [26] United States	Crossover 2 arms, 60 patients	52 weeks	Between 40 and 80 year with a clinical diagnosis of COPD (postalbuterol FEV1/FVC ratio < 0.70) and stable, without change in medications or exacerbation within the prior 4wk Current or ex-smokers with > 10 pack-years smoking history	Formoterol/Glycopyrronium (5/7.2 μg)	Placebo	CWRCE	Mean (95% CI) treatment difference, CWRCE: 55 s (20–90)
Tufvesson et al. 2021 [27] Sweden	Crossover 2 arms, 23 patients	n.a	FEV1 of 40– 80% of predicted normal (%pred) and a ratio of FEV1 to forced vital capacity (FVC) of ⩽0.7	Indacaterol/Glycopyrronium (110/50 μg)	Placebo	CWRCE	Mean (95% CI) treatment difference, CWRCE: 113 s (6–220)

The combinations of bronchodilators most commonly used as an intervention were tiotropium/olodaterol 5 µg/5 µg (7 studies), tiotropium/olodaterol 2.5 µg/5 µg (3 studies) and umeclidinium/vilanterol 62.5 µg/25 µg (3 studies). Comparators were placebo in 13 studies and monotherapy in 10 studies, using tiotropium 5 µg in 5 studies, olodaterol 5 µg in 1 study, umeclidinium 62.5 µg in 3 studies, umeclidinium 125 µg in 2 studies and vilanterol 25 µg in 2 studies. In two studies more than one monotherapy was used as a comparator.

### Risk of bias

The risk of bias was considered low for all domains evaluated except for blinding of the results and concealment of assignment domains, where the risk of bias was unclear for the majority of studies analysed (Additional file 1: Fig. A1).

### Effectiveness of the intervention

Table 2 shows a summary of meta-analysis comparisons for those variables of interest that estimated change from baseline.

**Table 2:** Summary of meta-analysis comparisons and main results in weighted mean differences (WMD) and standardised mean differences (SMD)

	LAMA/LABA comparator	Characteristics		Weighted results		Standardized results	
		Number of CT	N	MD	95% CI	MD	95% CI
ESWT	Placebo*	4	1730	31.75 s	16.03 s to 47.47 s	0.21	0.12 to 0.31
	Monotherapy	1	689	11.36%	− 0.03% to 22.74%	0.16	− 0.00 to 0.33
CWRCE	Placebo*	3	2466	72.45 s	46.77 s to 98.13 s	0.22	0.14 to 0.30
	Monotherapy	2	3398	24.23 s	− 0.86 s to 49.32 s	0.06	− 0.00 to 0.13
T_lim_ CWRT	Monotherapy*	1	38	43.80%	38.77% to 48.83%	5.42	3.99 to 6.86
6MWT	Placebo	1	125	11.87 m	− 9.32 m to 33.06 m	0.20	− 0.16 to 0.55
	Monotherapy*	4	634	9.77 m	1.22 m to 18.31 m	0.17	0.02 to 0.33
Steps/day	Placebo*	3	710	471.89 steps/day	206.08 steps/day to 737.71 steps/day	0.26	0.11 to 0.41
	Monotherapy**	3	521	398.48 steps/day	− 264.40 steps/day to 1061.36 steps/day	0.18	0.01 to 0.36
≥ 1.0–1.5 METs	Monotherapy*	2	315	− 9.93 min	− 17.91 min to − 1.95 min	− 0.30	− 0.53 to − 0.08
≥ 2.0 METs	Monotherapy*	3	645	5.59 min	2.13 min to 9.05 min	0.24	0.08 to 0.39
≥ 3.0 METs	Placebo***	2	612	7.73 min	3.07 min to 12.39 min	0.24	− 0.05 to 0.53
	Monotherapy*	2	315	2.60 min	0.74 min to 4.46 min	0.29	0.07 to 0.51
Energy expenditure	Placebo*	2	612	39.33 kcal/day	17.95 kcal/day to 60.71 kcal/day	0.28	0.12 to 0.44

^*^Statistically significant differences, both in WMD and SMD. **Statistically significant differences in SMD. ***Statistically significant differences in WMD

*CI* confidence interval, *CT* clinical trial, *CWRCE* constant work rate cycle ergometry, *ESWT* endurance shuttle walk test, *kcal* kilocalories, *LABA* long-acting beta-2 agonists, *LAMA* long-acting muscarinic antagonists, *m* meters, *MD* mean difference, *MET* metabolic equivalent of task, *min* minutes, *N* number of patients, *s* seconds, *SMD* standardized mean difference, *T*_*lim*_* CWRT* tolerance limit in constant work rate test, *WMD* weighted mean difference, *6MWT* 6-min walking test

#### Effectiveness of the intervention in exercise capacity

**Fig. 2:**
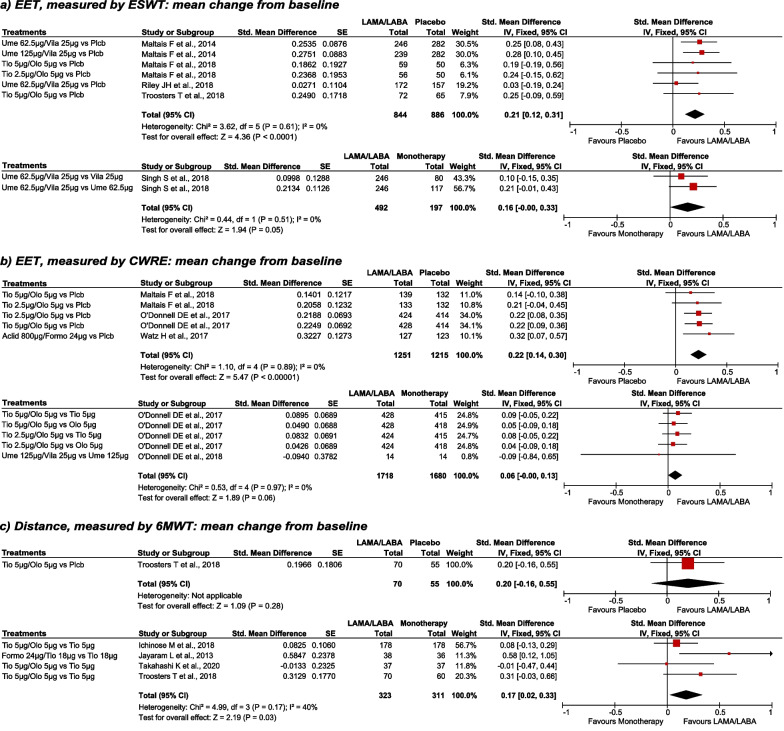
ESWT, CWRCE and 6MWT; Mean change from baseline, LAMA/LABA vs placebo and LAMA/LABA vs monotherapy. Fixed effects analysis model. *Aclid* aclidinium, *CI* confidence interval, *CWRCE* constant work rate cycle ergometry, *EET* exercise endurance time, *ESWT* endurance shuttle walk test, *Formo* formoterol, *IV* inverse variance, *LABA* long-acting beta-2 agonists, *LAMA* long-acting muscarinic antagonists, *Olo* olodaterol, *Plcb* placebo, *SE* standard error, *Std.* standardized, *Tio* Tiotropium, *Ume* umeclidinium, *Vila* vilanterol, *6MWT* 6-min walking test, *µm* microgram

The LAMA/LABA combination was associated with significantly better physical endurance than placebo, when evaluated by both ESWT and CWRCE (Fig. 2a, b). Compared with monotherapy, LAMA/LABA combinations showed favourable results, although, these results just failed to be statistically significant, both when evaluated by ESWT (SMD: 0.16; 95%CI: − 0.00 to 0.33) and by CWRCE (SMD: 0.06; 95%CI: − 0.00 to 0.13) (Fig. 2a, b).

**Fig. 3:**
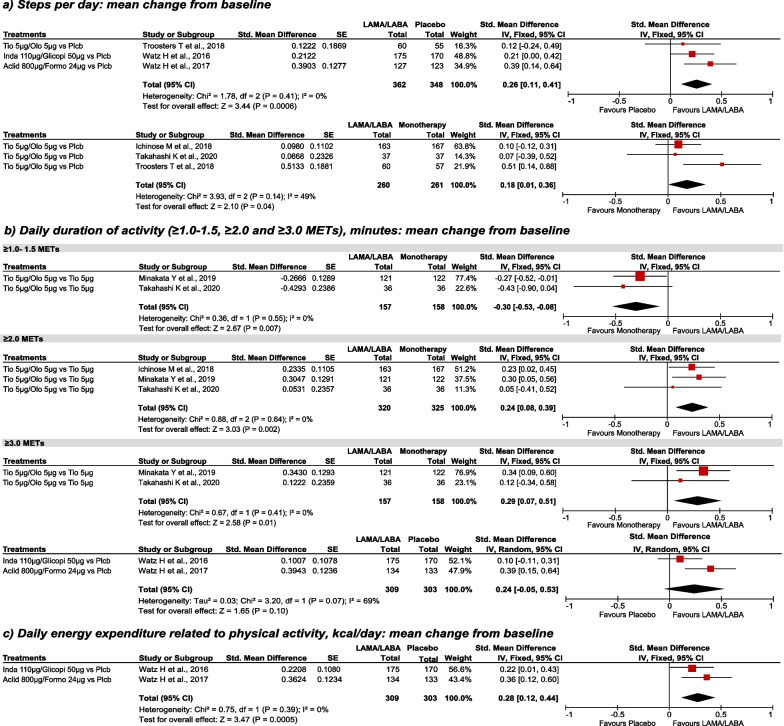
Steps/day, duration of activity and energy expenditure; mean change from baseline, LAMA/LABA vs placebo and vs monotherapy. Random effects analysis model (against placebo, ≥ 3.0 METs); fixed effects analysis model for other comparisons. *Aclid* aclidinium, *CI* confidence interval, *Formo* formoterol, *Glicopi* glycopyrronium, *Inda* indacaterol, *IV* inverse variance, *kcal* kilocalories, *LABA* long-acting beta-2 agonists, *LAMA* long-acting muscarinic antagonists, *MET* metabolic equivalent task, *Olo* olodaterol, *Plcb* placebo, *SE* standard error, *Std.* standardized, *Tio* Tiotropium, *µm* microgram

In the study by Maltais et al. a subgroup of patient of the TORRACTO study in which both CWRCE and ESTW were evaluated, results were consistent with previous publications and show significant superiority of LAMA/LABA combination vs. placebo in CWCRE (difference: 118.3 [95% CI: 45.9 to 190.8]; p = 0.0015), although these differences were not statistically significant in ESWT (difference: 76.3 [95% CI: − 2.8 to 155.4]; p = 0.0585) [22].

In Canto et al. [23] LAMA/LABA combination was compared to monotherapy, measuring the increase, in percentage, of the tolerance limit in constant work rate test. This comparison showed the superiority of LAMA/LABA combination against monotherapy with statistically significant differences (Table 2). By comparing LAMA/LABA against placebo, two studies estimated CWRCE after treatment (these studies were not designed to evaluate the change from baseline). In both studies a mean increase in exercise capacity was observed with LAMA/LABA versus placebo in COPD (55 s [95% CI: 20–90, p = 0.013] [26] y 113 s [95% CI: 6–220, p = 0.037] [27].

Regarding 6MWT, the mean difference of 11.87 mts. observed between LABA/LAMA and placebo in a single study (n = 125) did not reach statistical significance (Table 2); however, the meta-analysis of results of the 4 studies comparing LAMA/LABA with monotherapies (n = 634) showed significant differences in 6MWT in favour of LAMA/LABA combination (Table 2; Fig. 2c).

#### Effectiveness of the intervention in physical activity

When measured in steps per day, LAMA/LABA combinations were significantly superior to both placebo and monotherapy (Fig. 3a). Regarding daily duration activity, patients treated with LAMA/LABA combination reduced the duration of ≥ 1.0–1.5 METs activity than patients treated with monotherapy. On the other hand, for moderate physical activity, the results favoured LAMA/LABA therapy by increasing the duration of ≥ 2.0 METs activities. For vigorous physical activity (≥ 3.0 METs), LAMA/LABA therapy was superior to both monotherapy and placebo, although the latter results were not statistically significant when standardized under a random effects model (Fig. 3b). Daily activity-related energy expenditure was higher in the LAMA/LABA group than in the placebo group (Fig. 3c). Finally, more inactive patients (< 6000 steps/day) were observed in the placebo group than in the LAMA/LABA combination group (OR [95% CI]: 0.27 [0.14–0.51]; 1 study, N = 267) [15]. In Troosters et al. [12] walking intensity and walking time per day were also evaluated at week 12, results for average daily walking time mirrored those of steps per day and there was a small but significant increase in average daily walking intensity with SMBM plus placebo compared with baseline (1.97 vs. 1.90 m/s^2^, p = 0.006) and with SMBM + tiotropium/olodaterol (1.99 vs. 1.91 m/s^2^; p < 0.05) [12].

In Watz et al. [15] the D-PPAC questionnaire total score (LSM [95% CI]: 2.7 [1.3–4.1]; p = 0.0002), amount (3.4 [1.4–5.4]; p = 0.0008), and difficulty (2.3 [0.3–4.4]; p = 0.0258) domains improved significantly in the LAMA/LABA combination group versus placebo at week 4. At week 8, LAMA/LABA combination maintained the improvements seen after 4 weeks; however, the differences versus placebo were not statistically significant for either total score (1.2 [− 0.5 to 3.0]; p = 0.1710), amount (0.7 [− 2.1 to 3.4]; p = 0.6303) or difficulty (2.1 [− 0.4 to 4.5]; p = 0.0933) domains. In Troosters et al. [12] similar results were observed for LAMA/LABA combination and LAMA monotherapy vs. baseline at week 9 for difficulty and amount domain, and at week 12 for difficulty domain. No statistically improvements were shown when comparing LAMA/LABA vs. placebo, although numerically the combination showed better results in both domains at week 9 and 12 [12].

### Sensitivity analysis

Sensitivity analysis, stratified by study design in cases where heterogeneity was present, confirmed the LAMA/LABA combinations favorable results compared to monotherapy in 6MWT and steps per day. For vigorous physical activity (≥ 3.0 METs), LAMA/LABA therapy was superior to both monotherapies with a significant heterogeneity (I2 = 69%); due to the limited number of studies included in this analysis (n = 2), the sensitivity analysis was explained based on individual studies results. In Watz H et al. 2016 [21] LAMA/LABA combination was significantly better compared to monotherapy; whereas in Watz et al. 2017 [15] differences were not significant. Patients included in the Watz et al. 2016 [21] study appeared to have high durations of physical activity on entry (mean baseline values of 125 and 130 min per day), which were about 30% higher than in previous studies with similar COPD populations. This would suggest that the patients had limited opportunities to increase the duration of physical activity in their day-to-day lifestyle.

## Discussion

The results of this systematic review of RCTs indicate that exercise capacity and physical activity outcomes favoured LAMA/LABA combinations over placebo for ESTW, CWRE and steps per day; and over LAMA or LABA monotherapies for T_lim_ in CWRE, 6MWT and steps per day, where the differences were statistically significant. For LAMA/LABA versus placebo in 6MWT and versus monotherapy in ESTW and CWRE results favoured LAMA/LABA combinations, but the differences did not reach statistical significance.

The latest American Thoracic Society (ATS) guidelines on pharmacologic management of COPD recommend treating COPD patients who complain of dyspnoea or exercise intolerance with LAMA/LABA combination over LAMA or LABA monotherapies [29], as the combination of the two mechanisms of action effectively reduce the dynamic hyperinflation process characteristic in COPD patients, that usually limits their ability to exercise [6, 7, 30]. Several studies on COPD patients have associated low levels of physical activity and sedentary time with an increased frequency of exacerbations, hospitalizations, worse quality of life, and also an increased risk of death as a result of progressive ventilatory limitation, cardiac impairment, peripheral muscle, and psychological factors [3, 31, 32]. Increasing physical activity and its intensity in those patients may improve quality of life and reduce the loss of pulmonary function [33, 34].

Moreover, increasing the duration of low-intensity activity, instead of high-intensity activity, contributes to a lower risk of hospitalization in patients with moderate to severe COPD, which can be achieved with combined LAMA/LABA therapies [16, 25]. However, reaching better exercise capacity is no guarantee of physical activity improvements [12, 35]. Regarding this topic, in this meta-analysis, LAMA/LABA therapy significantly reduced the duration of 1.0–1.5 METs (sedentary time) and increased the durations of ≥ 2.0 METs (standing position or walking less than 55 m/min), and ≥ 3.0 MET (walking faster than 55 m/min). In general, our results provide proof of a significant reduction in sedentary time in patients with COPD who are administered LAMA/LABA compared to monotherapy.

The results observed in sedentary time were paralleled with significant improvements in daily walking time and in the intensity of walking in the D-PPAC questionnaire score where superiority of LAMA/LABA combinations over placebo was observed, as it was already noticed in the PHYSACTO and ACTIVATE studies. In the PHYSACTO study, significant differences in the questionnaire score between tiotropium monotherapy and the tiotropium/olodaterol combination were found [12], and in the ACTIVATE study between placebo and aclidinium/formoterol combination [15], indicating that LAMA/LABA combination improves the amount and level of intensity of physical activity in COPD patients.

In some observational studies [36–41] the use of tiotropium/olodaterol showed improvements in patient self-reported physical condition. Therapeutic success in the physical functioning score varied from 48.9% to 67.8%, with improved patient general condition as indicated by an improvement in Physician’s Global Evaluation scores between visits in these studies [36–41] and increased absolute physical functioning scores [36]. These results are consistent with those obtained in our meta-analysis, where tiotropium/olodaterol was the most frequent LAMA/LABA analysed versus monotherapy, used in five different studies [13, 14, 16, 25]. Also, tiotropium/olodaterol was compared to placebo in the study by Maltais et al. [20]. In general, LAMA/LABA combinations were superior to LAMA or LABA monotherapies. Differences were not significant when comparing LAMA/LABA versus monotherapy in ESWT or CWRCE tests, probably because there could be a threshold for bronchodilation to immediately translate into better exercise tolerance. It may be unrealistic to expect the same exercise benefit when adding a second bronchodilator to an existing one than when adding a bronchodilator to placebo [13]. These results agree with recent meta-analysis, which also concluded that LAMA/LABA combinations were more effective than LABA or LAMA monotherapy in terms of exercise capacity and symptoms [6, 42]. The meta-analysis by Di Marco et al. [6] showed weighted mean increase in endurance time of 78.4 s with LAMA/LABA, 72.6 s with LAMA monotherapy and 51 s with LABA monotherapy compared to placebo, and improvements in BORG scale score of -0.25 units with LAMA/LABA versus − 0.51 and − 0.45 with LABA and LAMA monotherapies respectively. The relative effect results of the meta-analysis by Calzetta et al. [42] also pointed LABA/LAMA as the combination significantly (P < 0.05) more effective than the LABA or LAMA alone and placebo in terms of improvement in endurance time (+ 43,  + 22 and + 60 s, respectively) and increase in inspiratory capacity as measure of reduction in lung hyperinflation (+ 107 ml, + 87 ml and + 229 ml, respectively), although these improvements were slightly lower than the ones observed by Di Marco et al. [6] as Calzzeta et al. point out [42]. The results of both meta-analyses are in line with our results, as in our analysis differences between LAMA/LABA versus placebo or monotherapy were also significant (LAMA/LABA vs placebo + 31.75–72.45 s, vs. monotherapy + 11.36% and + 24.23 s).

Besides pharmacological treatment, the ATS, the European Respiratory Society (ERS) and the Spanish guidelines for COPD agree on using non-pharmacological treatment as part of the comprehensive COPD patient care as increasing physical activity and reducing discomfort during physical activity requires a more integrated approach than only providing adequate bronchodilation and it should consider all aspects of the disease, including mental, physical and emotional health [43–47]. Besides, as hyperinflation is the main driver of the reduced physical activity in COPD patients, by combining effective bronchodilators with pulmonary rehabilitation pulmonary function will be optimized and gas trapping reduced, increasing patient’s exercise capacity [48–50]. Pulmonary rehabilitation includes exercise training, education and behavior change, aimed to improve the physical and psychological condition of COPD patients and to promote the long-term adherence to health-enhancing behaviours [47]. Before any actions are undertaken it is important to assess the initial level of physical activity in daily life as physical activity can be improved with the appropriate strategies in most COPD patients, and during all this process counselling or psychological programmes help supporting the change in behaviour that is needed for patients to be more active. Accordingly, the implementation of physical performance or muscle function/mass tests that correlate with objectively measured physical activity in clinical practice can be a good implementation to assess COPD patients’ level of daily physical activity, to identify those with severely reduced levels of physical activity (such the 6MWT, or the 30-s chair stand test), and establish an exercise plan taking into account personal needs, preferences and personal goals to go along with the pharmacological treatment [47, 51, 52]. The ESWT, CWRCE and 6MWT are the commonest test used to assess COPD patients’ level of physical activity; these are reliable tests to which patients respond and are familiarized with, they can be used in a multicentre trial setting, as they have good reproducibility and repeatability, and have an important intra class (IC) correlation and are significant predictors of mortality in COPD [14, 22, 53]. Particularly, ESWT has been reported to be more sensitive than other tests to therapeutic intervention in a systematic review, where protocol variations significantly affected performance in several studies [53].

This SRL and meta-analysis has some limitations, the main one is the existing differences between the studies on variables used to measure physical activity which, in some cases, makes comparison difficult. Furthermore, it should be taken into account that in some analyses different LAMA/LABA combinations were compared with different LAMA or LABA monotherapies, and also outcomes evaluation times were different between studies, ranging from 3 to 12 weeks. Another limitation is that statistical heterogeneity was high in some comparison, limiting the validity and the generalizability of these results. Despite these limitations, the use of LAMA/LABA consistently improves exercise capacity and physical activity compared with placebo or monotherapy in most outcomes and combinations analysed. On the other hand, our study has the following strengths: a reasonable number of studies and patients available and their rigorous methodological quality, as none of the studies included showed high risk of bias in any item.

## Conclusion

In conclusion, our review showed that LAMA/LABA combination therapy was superior to placebo and monotherapy in terms of evaluating exercise capacity and physical activity in patients with COPD in almost every comparison. Enhancing physical activity and exercise capacity in COPD patients might lead to improve their quality of life and minimize the burden of the disease.

## Supplementary Information

**Additional file 1: Fig A1. **Bias risk assessment.

**Additional file 2: Table A1**. Search strategy in MEDLINE (through PubMed), CENTRAL and EMBASE.

## Data Availability

This manuscript is an SLR and the data used are the ones available at the included publications, thus this sections is not applicable.

## Abbreviations

6MWT: 6-Minute walking test
ATS: American Thoracic Society
CI: Confidence intervals
COPD: Chronic obstructive pulmonary disease
CWRCE: Constant Work Rate Cycle Ergometry
D-PPAC: Daily PROactive Physical Activity COPD questionnaire
ERS: European Respiratory Society
ESWT: Endurance Shuttle Walk Test
FEV1: Forced expiratory volume at 1 s
FVC: Forced vital capacity
GOLD: Global Initiative for Chronic Obstructive Lung Disease
LABA: Long-acting beta-2 agonists
LAMA: Long-acting muscarinic antagonists
METs: Metabolic equivalent of task
OR: Odds ratio
PRISMA: Preferred Reporting Items for Systematic Reviews and Meta-analyses Statement
PRO: Patient-reported outcome
SLR: Systematic literature review
SMD: Standardized mean differences
T_lim_: Tolerance limit
WMD: Weighted mean differences

## References

[1] Global Initiative for Chronic Obstructive Lung Disease. Global Strategy for Diagnosis, Management and Prevention of COPD. The Global Initiative for Chronic Obstructive Lung Diseases (GOLD). 2020.

[2] Tekerlek H, Cakmak A, Calik-Kutukcu E, Arikan H, Inal-Ince D, Saglam M (2020). Exercise capacity and activities of daily living are related in patients with chronic obstructive pulmonary disease. Arch Bronconeumol.

[3] Waschki B, Kirsten A, Holz O, Müller K-C, Meyer T, Watz H (2011). Physical activity is the strongest predictor of all-cause mortality in patients with COPD. Chest.

[4] Mendoza L, de Oca MM, López Varela MV, Casas A, Ramírez-Venegas A, López A (2021). Physical activity levels and associated factors in a Latin American COPD population of patients. The LASSYC Study. COPD J Chronic Obstr Pulmon Dis..

[5] Garcia-Rio F, Lores V, Mediano O, Rojo B, Hernanz A, López-Collazo E (2009). Daily physical activity in patients with chronic obstructive pulmonary disease is mainly associated with dynamic hyperinflation. Am J Respir Crit Care Med.

[6] di Marco F, Sotgiu G, Santus P, O’Donnell DE, Beeh K-M, Dore S (2018). Long-acting bronchodilators improve exercise capacity in COPD patients: a systematic review and meta-analysis. Respir Res.

[7] Anzueto A, Miravitlles M (2018). Considerations for the correct diagnosis of COPD and its management with bronchodilators. Chest.

[8] Rossi A, Aisanov Z, Avdeev S, di Maria G, Donner CF, Izquierdo JL (2015). Mechanisms, assessment and therapeutic implications of lung hyperinflation in COPD. Respir Med.

[9] Moher D, Shamseer L, Clarke M, Ghersi D, Liberati A, Petticrew M (2015). Preferred reporting items for systematic review and meta-analysis protocols (PRISMA-P) 2015 statement. Syst Rev.

[10] Gimeno-Santos E, Raste Y, Demeyer H, Louvaris Z, de Jong C, Rabinovich RA (2015). The PROactive instruments to measure physical activity in patients with chronic obstructive pulmonary disease. Eur Respir J.

[11] Higgins JPT, Thomas J, Chandler J, Cumpston M, Li T, Page MJ WV. Cochrane Handbook for Systematic Reviews of Interventions version 6.1. 2020.

[12] Troosters T, Maltais F, Leidy N, Lavoie KL, Sedeno M, Janssens W (2018). Effect of bronchodilation, exercise training, and behavior modification on symptoms and physical activity in chronic obstructive pulmonary disease. Am J Respir Crit Care Med.

[13] O’Donnell DE, Casaburi R, Frith P, Kirsten A, de Sousa D, Hamilton A (2017). Effects of combined tiotropium/olodaterol on inspiratory capacity and exercise endurance in COPD. Eur Respir J.

[14] Ichinose M, Minakata Y, Motegi T, Ueki J, Gon Y, Seki T (2018). Efficacy of tiotropium/olodaterol on lung volume, exercise capacity, and physical activity. Int J Chron Obstruct Pulmon Dis.

[15] Watz H, Troosters T, Beeh KM, Garcia Aymerich J, Paggiaro P, Molins E (2017). ACTIVATE: the effect of aclidinium/formoterol on hyperinflation, exercise capacity, and physical activity in patients with COPD. Int J Chron Obstruct Pulmon Dis.

[16] Minakata Y, Motegi T, Ueki J, Gon Y, Nakamura S, Anzai T (2019). Effect of tiotropium/olodaterol on sedentary and active time in patients with COPD: post hoc analysis of the VESUTO^®^ study. Int J Chron Obstruct Pulmon Dis.

[17] Singh S, Maltais F, Tombs L, Fahy WA, Vahdati-Bolouri M, Locantore N (2018). Relationship between exercise endurance and static hyperinflation in a post hoc analysis of two clinical trials in patients with COPD. Int J Chron Obstruct Pulmon Dis.

[18] Riley JH, Kalberg CJ, Donald A, Lipson DA, Shoaib M, Tombs L (2018). Effects of umeclidinium/vilanterol on exercise endurance in COPD: a randomised study. ERJ Open Res.

[19] O’Donnell DE, Elbehairy AF, Faisal A, Neder JA, Webb KA (2018). Sensory-mechanical effects of a dual bronchodilator and its anticholinergic component in COPD. Respir Physiol Neurobiol.

[20] Maltais F, O’Donnell D, GáldizIturri JB, Kirsten A-M, Singh D, Hamilton A (2018). Effect of 12 weeks of once-daily tiotropium/olodaterol on exercise endurance during constant work-rate cycling and endurance shuttle walking in chronic obstructive pulmonary disease. Ther Adv Respir Dis.

[21] Watz H, Mailänder C, Baier M, Kirsten A (2016). Effects of indacaterol/glycopyrronium (QVA149) on lung hyperinflation and physical activity in patients with moderate to severe COPD: a randomised, placebo-controlled, crossover study (The MOVE Study). BMC Pulm Med.

[22] Maltais F, O’Donnell DE, Hamilton A, Zhao Y, Casaburi R (2020). Comparative measurement properties of constant work rate cycling and the endurance shuttle walking test in COPD: the TORRACTO^®^ clinical trial. Ther Adv Respir Dis.

[23] Canto ND, Ribeiro JP, Neder JA, Chiappa GR (2012). Addition of tiotropium to formoterol improves inspiratory muscle strength after exercise in COPD. Respir Med.

[24] Jayaram L, Wong C, McAuley S, Rea H, Zeng I, O’Dochartaigh C (2013). Combined therapy with tiotropium and formoterol in chronic obstructive pulmonary disease: effect on the 6-minute walk test. COPD J Chron Obstruct Pulmon Dis..

[25] Takahashi K, Uchida M, Kato G, Takamori A, Kinoshita T, Yoshida M (2020). First-line treatment with tiotropium/olodaterol improves physical activity in patients with treatment-naïve chronic obstructive pulmonary disease. Int J Chron Obstruct Pulmon Dis.

[26] Stringer WW, Porszasz J, Cao M, Rossiter HB, Siddiqui S, Rennard S (2021). The effect of long-acting dual bronchodilator therapy on exercise tolerance, dynamic hyperinflation, and dead space during constant work rate exercise in COPD. J Appl Physiol.

[27] Tufvesson E, Radner F, Simonsen A, Papapostolou G, Jarenbäck L, Jönsson S (2021). A new protocol for exercise testing in COPD; improved prediction algorithm for W MAX and validation of the endurance test in a placebo-controlled double bronchodilator study. Ther Adv Respir Dis..

[28] Maltais F, Singh S, Donald AC, Crater G, Church A, Goh AH (2014). Effects of a combination of umeclidinium/vilanterol on exercise endurance in patients with chronic obstructive pulmonary disease: two randomized, double-blind clinical trials. Ther Adv Respir Dis.

[29] Nici L, Mammen MJ, Charbek E, Alexander PE, Au DH, Boyd CM (2020). Pharmacologic management of chronic obstructive pulmonary disease. Am J Respir Crit Care Med.

[30] Anzueto A, Miravitlles M (2018). The role of fixed-dose dual bronchodilator therapy in treating COPD. Am J Med.

[31] Miravitlles M, Cantoni J, Naberan K (2014). Factors associated with a low level of physical activity in patients with chronic obstructive pulmonary disease. Lung.

[32] Koreny M, Demeyer H, Benet M, Arbillaga-Etxarri A, Balcells E, Barberan-Garcia A (2021). Patterns of physical activity progression in patients with COPD. Arch Bronconeumol.

[33] Demeyer H, Donaire-Gonzalez D, Gimeno-Santos E, Ramon MA, de Battle J, Benet M (2019). Physical activity is associated with attenuated disease progression in chronic obstructive pulmonary disease. Med Sci Sports Exerc.

[34] Waschki B, Kirsten AM, Holz O, Mueller K-C, Schaper M, Sack A-L (2015). Disease progression and changes in physical activity in patients with chronic obstructive pulmonary disease. Am J Respir Crit Care Med.

[35] Sievi NA, Brack T, Brutsche MH, Frey M, Irani S, Leuppi JD (2020). “Can do, don’t do” are not the lazy ones: a longitudinal study on physical functioning in patients with COPD. Respir Res.

[36] Glaab T, Sauer R, Hänsel M, Rubin RA, Frey M, Buhl R (2016). Impact of tiotropium + olodaterol on physical functioning in COPD: results of an open-label observational study. Int J Chron Obstruct Pulmon Dis..

[37] Steinmetz K-O, Abenhardt B, Pabst S, Hänsel M, Kondla A, Bayer V (2019). Assessment of physical functioning and handling of tiotropium/olodaterol Respimat^®^ in patients with COPD in a real-world clinical setting. Int J Chron Obstruct Pulmon Dis.

[38] Valipour A, Tamm M, Kociánová J, Bayer V, Sanzharovskaya M, Medvedchikov A (2019). Improvement in self-reported physical functioning with tiotropium/olodaterol in central and Eastern European COPD Patients. Int J Chron Obstruct Pulmon Dis.

[39] Molina París J, Alonso Hernández PM, Díez García JA, Gonzalez Uribe-Etxebarria I, Yelo García J, Galera Llorca J (2020). Assessment of physical functioning in patients with chronic obstructive pulmonary disease (COPD) requiring long-acting dual bronchodilation in routine clinical practice. Med Familia Semergen..

[40] Carone M, Pennisi A, D’Amato M, Donati AF, Ricci A, Scognamillo C (2020). Physical functioning in patients with chronic obstructive pulmonary disease treated with tiotropium/olodaterol respimat in routine clinical practice in Italy. Pulm Ther.

[41] Spielmanns M, Tamm M, Schildge S, Valipour A (2021). Swiss experience in therapy with dual bronchodilation in chronic obstructive pulmonary disease in relation to self-reported physical functionality. J Clin Med Res.

[42] Calzetta L, Ora J, Cavalli F, Rogliani P, O’Donnell DE, Cazzola M (2017). Impact of LABA/LAMA combination on exercise endurance and lung hyperinflation in COPD: a pair-wise and network meta-analysis. Respir Med.

[43] Nici L, ZuWallack R (2012). An official American Thoracic Society workshop report: the integrated care of the COPD patient. Proc Am Thorac Soc.

[44] Spruit MA, Singh SJ, Garvey C, ZuWallack R, Nici L, Rochester C (2013). An Official American Thoracic Society/European Respiratory Society Statement: key concepts and advances in pulmonary rehabilitation. Am J Respir Crit Care Med.

[45] Miravitlles M, Calle M, Molina J, Almagro P, Gómez J-T, Trigueros JA (2022). Spanish COPD Guidelines (GesEPOC) 2021: updated Pharmacological treatment of stable COPD. Arch Bronconeumol.

[46] Cosío BG, Hernández C, Chiner E, Gimeno-Santos E, Pleguezuelos E, Seijas N, et al. [Translated article] Spanish COPD Guidelines (GesEPOC 2021): Non-pharmacological Treatment Update. Arch Bronconeumol. 2022;58:345–51. 10.1016/j.arbres.2021.08.010 35312554

[47] Maltais F, de la Hoz A, Casaburi R, O’Donnell D (2021). Effects of tiotropium/olodaterol on activity-related breathlessness, exercise endurance and physical activity in patients with COPD: narrative review with meta-/pooled analyses. Adv Ther.

[48] Wouters EFM, Wouters BBREF, Augustin IML, Houben-Wilke S, Vanfleteren LEGW, Franssen FME (2018). Personalised pulmonary rehabilitation in COPD. Eur Respir Rev.

[49] Güell M-R, Cejudo P, Ortega F, Puy MC, Rodríguez-Trigo G, Pijoan JI (2017). Benefits of long-term pulmonary rehabilitation maintenance program in patients with severe chronic obstructive pulmonary disease. Three-year follow-up. Am J Respir Crit Care Med.

[50] Pleguezuelos E, Esquinas C, Moreno E, Guirao L, Ortiz J, Garcia-Alsina J (2016). Muscular dysfunction in COPD: systemic effect or deconditioning?. Lung.

[51] Matkovic Z, Tudoric N, Cvetko D, Esquinas C, Rahelic D, Zarak M (2020). Easy to perform physical performance tests to identify COPD patients with low physical activity in clinical practice. Int J Chron Obstruct Pulmon Dis.

[52] Demeyer H, Mohan D, Burtin C, Vaes A, Heasley M, Bowler RP (2021). Objectively measured physical activity in patients with COPD: recommendations from an international task force on physical activity. Chron Obstruct Pulmon Dis J COPD Found.

[53] Fotheringham I, Meakin G, Punekar Y, Riley J, Cockle S, Singh S (2015). Comparison of laboratory- and field-based exercise tests for COPD: a systematic review. Int J Chron Obstruct Pulmon Dis..

