# Supplementary material for: Exercise capacity and physical activity in COPD patients treated with a LAMA/LABA combination: a systematic review and meta-analysis
Source: Respir Res. 2022 Dec 15;23:347. doi: 10.1186/s12931-022-02268-3 (PMC9753337; doi:10.1186/s12931-022-02268-3)
Supplement: Supplementary file 1 — Additional file 1: Fig A1. Bias risk assessment. [file 12931_2022_2268_MOESM1_ESM.pdf]

| | Random sequence generation (selection bias) | Allocation concealment (selection bias) | Blinding of participants and personnel (performance bias) | Blinding of outcome assessment (detection bias) | Incomplete outcome data (attrition bias) | Selective reporting (reporting bias) | Other bias |
|---|---|---|---|---|---|---|---|
| Canto., 2012 | + | ? | + | ? | + | + | + |
| Ichinose., 2018 | + | + | + | + | + | + | + |
| Jayaram., 2013 | + | − | + | ? | + | + | + |
| Maltais., 2014 | + | + | + | ? | ? | + | + |
| Maltais., 2018 | + | ? | + | ? | − | + | + |
| Maltais., 2020 | + | ? | + | ? | + | + | + |
| Minakata., 2019 | + | + | + | + | + | + | + |
| O'Donell., 2017 | + | ? | + | ? | + | + | + |
| O'Donnell., 2018 | + | ? | + | ? | ? | + | + |
| Riley., 2018 | + | + | + | ? | + | + | + |
| Singh., 2018 | + | ? | + | ? | ? | + | + |
| Stringer., 2021 | + | + | + | ? | + | + | + |
| Takahashi et al., 2020 | + | − | − | ? | + | + | + |
| Troosters., 2018 | + | ? | − | ? | + | + | + |
| Tufvesson., 2021 | + | ? | + | ? | + | − | ? |
| Watz., 2016 | + | ? | + | + | + | + | + |
| Watz., 2017 | + | ? | + | ? | + | + | + |
