# Supplementary material for: Exercise capacity and physical activity in COPD patients treated with a LAMA/LABA combination: a systematic review and meta-analysis
Source: Respir Res. 2022 Dec 15;23:347. doi: 10.1186/s12931-022-02268-3 (PMC9753337; doi:10.1186/s12931-022-02268-3)
Supplement: Supplementary file 2 — Additional file 2: Table A1. Search strategy in MEDLINE (through PubMed), CENTRAL and EMBASE. [file 12931_2022_2268_MOESM2_ESM.docx]

ADDITIONAL FILE 2

Table A. 1. Search strategy in MEDLINE (through PubMed), CENTRAL and EMBASE

| PUBMED | | |
| --- | --- | --- |
| Search | Concept | Query |
| **1** | **COPD** | (EPOC) OR COPD) OR "enfermedad pulmonar obstructiva cronica") OR "chronic obstructive pulmonary disease") |
| **2** | **LAMA** | (LAMA) OR "long acting muscarinic antagonist") OR "long-acting muscarinic antagonist") OR "antagonistas muscarinicos de accion prolongada") OR glicopirronio) OR glycopyrronium) OR aclidinio) OR aclidinium) OR tiotropio) OR tiotropium) OR umeclidinium) OR umeclidinio))) OR "long acting muscarinic antagonists") OR "long-acting muscarinic antagonists") OR "antagonista muscarínico de accion prolongada")) |
| **3** | **LABA** | ("long-acting beta2-adrenoceptor agonist") OR "long acting beta2-adrenoceptor agonist")) OR ((((((((((LABA) OR "long acting beta agonist") OR "long-acting beta agonist") OR "agonistas beta2 de accion prolongada") OR "agonista beta2 de accion larga") OR vilanterol) OR indacaterol) OR formoterol) OR olodaterol))) |
| **4** | **LAMA/LABA** | #2 AND #3 |
| **5** | **Exercise capacity and physical activity** | ("actividad física") OR "actividades físicas") OR "physical activity") OR "physical activities") OR "ejercicio físico") OR "physical exercise") OR "ejercicios físicos") OR "physical exercises") OR "borg scale") OR "escala de borg") OR "PF 10") OR "PF-10") OR "Physical Functioning") OR "funcionalidad física") OR step OR steps) OR paso OR pasos) OR 6MWT) OR 6MWD) OR "6 minutes walking test") OR "6 minutes walking distance") OR "walking test") OR "prueba 6 minutos marcha") OR "prueba seis minutos marcha") OR "Prueba de caminata de seis minutos") OR "Prueba de caminata de 6 minutos") OR "endurance test") OR "endurance shuttle walking") OR "constant work-rate cycling") OR "exercise endurance time") OR EET) OR "cycle endurance test") OR CET) OR "Prueba de esfuerzo") OR ergometría) OR deporte OR deportes) OR sport OR sports)) OR Esfuerzo*) OR Resistencia*) OR Distancia*) OR distance*) OR ((((((("Volumen Espiratorio forzado") OR VEMS) OR "Capacidad Vital Forzada") OR CVF)) OR "capacidad inspiratoria") OR CI)) OR "Inspiratory capacity") OR IC) OR "Forced Expiratory Volume") OR FEV) OR FEV1) OR "Forced vital capacity") OR FVC) OR endurance*)) OR exercise*) OR ejercicio*) |
| **6** | **Exercise capacity and physical activity outcomes in COPD patients treated with LAMA/LABA bronchodilators** | #1 AND #4 AND #5 |
| **CENTRAL** | | |
| **1** | **COPD** | (EPOC OR COPD) OR ("enfermedad pulmonar obstructiva cronica" OR "chronic obstructive pulmonary disease") |
| **2** | **LAMA** | LAMA OR ("long acting muscarinic antagonist" OR "long-acting muscarinic antagonist" OR "antagonistas muscarinicos de accion prolongada") OR (glicopirronio OR glycopyrronium) OR (aclidinio OR aclidinium) OR (tiotropio OR tiotropium) OR (umeclidinium OR umeclidinio) OR ("long acting muscarinic antagonists" OR "long-acting muscarinic antagonists") OR ("antagonista muscarínico de accion prolongada") |
| **3** | **LABA** | ("long-acting beta2-adrenoceptor agonist" OR "long acting beta2-adrenoceptor agonist") OR LABA OR ("long acting beta agonist" OR "long-acting beta agonist" OR "agonistas beta2 de accion prolongada" OR "agonista beta2 de accion larga") OR (vilanterol OR indacaterol OR formoterol OR olodaterol) |
| **4** | **LAMA/LABA** | #2 AND #3 |
| **5** | **Exercise capacity and physical activity** | "actividad física" OR "actividades físicas" OR "physical activity" OR "physical activities") OR ("ejercicio físico" OR "physical exercise" OR "ejercicios físicos" OR "physical exercises") OR ("borg scale" OR "escala de borg") OR ("PF 10" OR "PF-10") OR ("Physical Functioning" OR "funcionalidad física") OR (step OR steps OR paso OR pasos) OR (6MWT OR 6MWD OR "6 minutes walking test" OR "6 minutes walking distance" OR "walking test" OR "prueba 6 minutos marcha" OR "prueba seis minutos marcha" OR "Prueba de caminata de seis minutos" OR "Prueba de caminata de 6 minutos") OR ("endurance test" OR "endurance shuttle walking" OR "constant work-rate cycling" OR "exercise endurance time" OR EET OR "cycle endurance test" OR CET) OR ("Prueba de esfuerzo") OR ergometría OR (deporte OR deportes OR sport OR sports) OR Esfuerzo* OR Resistencia* OR Distancia* OR distance* OR ("Volumen Espiratorio forzado" OR VEMS) OR ("Capacidad Vital Forzada" OR CVF) OR ("capacidad inspiratoria" OR CI OR "Inspiratory capacity" OR IC) OR ("Forced Expiratory Volume" OR FEV OR FEV1 OR "Forced vital capacity" OR FVC) OR endurance* OR exercise* OR ejercicio* |
| **6** | **Exercise capacity and physical activity outcomes in COPD patients treated with LAMA/LABA bronchodilators** | #1 AND #4 AND #5 |
| **7** | **Time restriction** | #1 AND #4 AND #5 publication date in Cochrane Library between Jan 2012 and Dec 2021 |
| **8** | **Time restriction** | #7 publication date in clinical trials between 2012 and 2021 |
| **EMBASE** | | |
| **1** | **COPD** | (EPOC OR COPD OR "enfermedad pulmonar obstructiva cronica" OR "chronic obstructive pulmonary disease").mp. [mp=title, abstract, heading wors, drug trade number, original title, device manufacturer, drug manufacturer, device trade name, keyword heading word, floating subheading word, candidate term word] |
| **2** | **Inclusion criteria restriction** | Limit 1 to (embase randomized controlled trial and yr=2012-2021 and article and (adult<18 to 64 years> or aged <65+ years>)) |
| **3** | **LAMA** | (LAMA OR "long acting muscarinic antagonist" OR "long-acting muscarinic antagonist" OR "antagonistas muscarinicos de accion prolongada" OR glicopirronio OR glycopyrronium OR aclidinio OR aclidinium OR tiotropio OR tiotropium OR umeclidinium OR umeclidinio OR "long acting muscarinic antagonists" OR "long-acting muscarinic antagonists" OR "antagonista muscarínico de accion prolongada").mp. [mp=title, abstract, heading wors, drug trade number, original title, device manufacturer, drug manufacturer, device trade name, keyword heading word, floating subheading word, candidate term word] |
| **4** | **Inclusion criteria restriction** | Limit 3 to (embase randomized controlled trial and yr=2012-2021 and article and (adult<18 to 64 years> or aged <65+ years>)) |
| **5** | **LABA** | ("long-acting beta2-adrenoceptor agonist" OR "long acting beta2-adrenoceptor agonist" OR LABA OR ("long acting beta agonist" OR "long-acting beta agonist" OR "agonistas beta2 de accion prolongada" OR "agonista beta2 de accion larga" OR vilanterol OR indacaterol OR formoterol OR olodaterol). mp. [mp=title, abstract, heading wors, drug trade number, original title, device manufacturer, drug manufacturer, device trade name, keyword heading word, floating subheading word, candidate term word] |
| **6** | **Inclusion criteria restriction** | Limit 5 to (embase randomized controlled trial and yr=2012-2021 and article and (adult<18 to 64 years> or aged <65+ years>)) |
| **7** | **LAMA/LABA** | #4 and #6 |
| **8** | **Exercise capacity and physical activity** | ("actividad física" OR "actividades físicas" OR "physical activity" OR "physical activities" OR "ejercicio físico" OR "physical exercise" OR "ejercicios físicos" OR "physical exercises" OR "borg scale" OR "escala de borg" OR "PF 10" OR "PF-10" OR "Physical Functioning" OR "funcionalidad física" OR step OR steps OR paso OR pasos OR 6MWT OR 6MWD OR "6 minutes walking test" OR "6 minutes walking distance" OR "walking test" OR "prueba 6 minutos marcha" OR "prueba seis minutos marcha" OR "Prueba de caminata de seis minutos" OR "Prueba de caminata de 6 minutos" OR "endurance test" OR "endurance shuttle walking" OR "constant work-rate cycling" OR "exercise endurance time" OR EET OR "cycle endurance test" OR CET OR "Prueba de esfuerzo" OR ergometría OR (deporte OR deportes OR sport OR sports)OR Esfuerzo OR Resistencia OR Distancia OR distance OR ("Volumen Espiratorio forzado" OR VEMS OR "Capacidad Vital Forzada" OR CVF OR "capacidad inspiratoria" OR CI OR "Inspiratory capacity" OR IC OR "Forced Expiratory Volume" OR FEV OR FEV1 OR "Forced vital capacity" OR FVC OR endurance OR exercise OR ejercicio). mp. [mp=title, abstract, heading wors, drug trade number, original title, device manufacturer, drug manufacturer, device trade name, keyword heading word, floating subheading word, candidate term word] |
| **9** | **Inclusion criteria restriction** | Limit 8 to (embase randomized controlled trial and yr=2012-2021 and article and (adult<18 to 64 years> or aged <65+ years>)) |
| **10** | **Exercise capacity and physical activity outcomes in COPD patients treated with LAMA/LABA bronchodilators** | 2 and 7 and 9 |
